# Epidemiology, Etiology, Diagnosis, and Management of Placenta Accreta

**DOI:** 10.1155/2012/873929

**Published:** 2012-05-07

**Authors:** Gali Garmi, Raed Salim

**Affiliations:** ^1^Department of Obstetrics and Gynecology, Emek Medical Centre, 18101 Afula, Israel; ^2^Rappaport Faculty of Medicine, Technion-Israel Institute of Technology, 31096 Haifa, Israel

## Abstract

Placenta accreta is a severe pregnancy complication and is currently the most common indication for peripartum hysterectomy. It is becoming an increasingly common complication mainly due to the increasing rate of cesarean delivery. Main risk factor for placenta accreta is a previous cesarean delivery particularly when accompanied with a coexisting placenta previa. Antenatal diagnosis seems to be a key factor in optimizing maternal outcome. Diagnosis can be achieved by ultrasound in the majority of cases. Women with placenta accreta are usually delivered by a cesarean section. In order to avoid an emergency cesarean and to minimize complications of prematurity it is acceptable to schedule cesarean at 34 to 35 weeks. A multidisciplinary team approach and delivery at a center with adequate resources, including those for massive transfusion are both essential to reduce neonatal and maternal morbidity and mortality. The optimal management after delivery of the neonate is vague since randomized controlled trials and large cohort studies are lacking. Cesarean hysterectomy is probably the preferable treatment. In carefully selected cases, when fertility is desired, conservative management may be considered with caution. The current review discusses the epidemiology, predisposing factors, pathogenesis, diagnostic methods, clinical implications and management options of this condition.

## 1. Introduction

Placenta accreta occurs when the chorionic villi invade the myometrium abnormally. It is divided into three grades based on histopathology: placenta accreta where the chorionic villi are in contact with the myometrium, placenta increta where the chorionic villi invade the myometrium, and placenta percreta where the chorionic villi penetrate the uterine serosa [1].

Placenta accreta is considered a severe pregnancy complication that may be associated with massive and potentially life-threatening intrapartum and postpartum hemorrhage [2]. It has become the leading cause of emergency hysterectomy [3]. Maternal morbidity had been reported to occur in up to 60% and mortality in up to 7% of women with placenta accreta. In addition, the incidence of perinatal complications is also increased mainly due to preterm birth and small for gestational age fetuses [4–7].

Once a rare occurrence, placenta accreta is becoming an increasingly common complication of pregnancy, mainly due to the increasing rate of cesarean delivery over the past 50 years [8]. In view of the fact that the indications for cesarean delivery seem to be steadily expanding, including cesarean delivery on maternal request, the incidence of placenta accreta is likely to continue to increase [9]. Wu et al. reported an incidence of 1 : 533 births for the period from 1982 to 2002, much greater than previous reports ranging from 1 : 4027 to 1 : 2510 births in the 1970s to 1980s, suggesting that this increase is mainly the result of the increasing rate of cesarean delivery [10]. Several risk factors for placenta accreta have been reported, including a previous cesarean delivery particularly when accompanied with a coexisting placenta previa. Increasing numbers of prior cesarean deliveries exponentially increase the risk of placenta accreta [10–14]. The exact pathogenesis of placenta accreta is unknown. A proposed hypothesis includes a maldevelopment of decidua, excessive trophoblastic invasion, or a combination of both [15]. Defective decidualization, abnormal maternal vascular remodeling, excessive trophoblastic invasion, or combinations thereof are considered to be the consequences of previous instrumentation [16]. Due to the high morbidity associated with this condition, accurate preoperative diagnosis of placenta accreta plays a crucial role in the management of these situations. Antenatal sonography is used to support the diagnosis and guide clinical management leading probably to favorable outcomes [17]. It has long been accepted that the definitive treatment of placenta accreta is hysterectomy [18, 19]. Conservative options which include leaving all or part of the placenta in situ when fertility preservation is desired had also been suggested [20, 21]. Several adjuvant techniques have been proposed alongside surgery. These included methotrexate treatment and/or placement of preoperative internal iliac artery balloon catheters for occlusion and/or arterial embolization to reduce intraoperative blood loss and transfusion requirements [1].

## 2. Epidemiology

Once a rare occurrence, placenta accreta is becoming an increasingly common complication of pregnancy, likely related to the increasing rate of cesarean delivery over the last five decades [8]. Placenta accreta occurs in approximately 1 : 1000 deliveries with a reported range from 0.04% rising up to 0.9% [10, 11, 22]. Differences in definition and study population may account for this wide range. The bladder is the most frequently involved extrauterine organ when there is a placenta percreta. Placenta percreta that invades the urinary bladder is associated with a substantial morbidity and mortality [23].

The median maternal age is around 34 years and the median parity is 2.5. The risk of developing placenta accreta increases with the number of previous cesarean deliveries. These range from 2% among women with a placenta previa only to 39–60% among women with accompanied two or more prior cesarean deliveries. Up to 88% of the women have concomitant placenta previa [11, 24]. Risk factors other than a previous cesarean include submucous myoma, previous curettage, Asherman's syndrome, advanced maternal age, grandmultiparity, smoking, and chronic hypertension [14]. In addition, Alanis et al. reviewed 72 cases of placenta accreta that were treated conservatively. Among 15% of women who had a subsequent pregnancy, 18% developed a repeated placenta accreta [25], so that prior placenta accreta is probably a major risk factor as well.

## 3. Pathogenesis

The exact pathogenesis of placenta accreta is unknown. Generally, placenta accreta has been diagnosed on hysterectomy specimens when an area of accretion showed chorionic villi in direct contact with the myometrium and an absence of decidua [16, 26]. This finding may be focal in some cases while the decidua is present in areas adjacent to the foci of accreta. This decidual maldevelopment in placenta accreta is usually associated with previous instrumentation as in the case of prior cesarean sections or uterine curettages [27].

A proposed hypothesis includes a maldevelopment of decidua, excessive trophoblastic invasion, or a combination of both. Tseng and Chou hypothesized that the abnormal expression of growth, angiogenesis, and invasion-related factors in the trophoblast populations are the main factors responsible for the occurrence of placenta accreta [27]. Additionally, Cohen et al. reported that the cytotrophoblast secretes factors that favor invasion, whereas decidua seems not to have a major role in regulating cytotrophoblast invasion in vitro [28]. On the contrary Earl et al. reported that the immunophenotype of extravillous trophoblastic populations in placenta accreta is identical to that seen in normal placenta, suggesting that it is unlikely that overactive trophoblastic invasion plays a major role in the pathogenesis of placenta accreta, and the absence of decidua is of greater importance in the pathogenesis [29]. Data from Tantbirojn et al. explained invasion of larger vessels in the outer myometrium and near the serosa to be determined by access rather than a preexisting defect in trophoblastic growth that would produce uncontrolled invasion through the entire depth of the myometrium in cases of accreta [16]. They propose that increta and percreta more likely arise due to dehiscence of a scar, which gives cells from the trophoblast column better access to large outer myometrial vessels [16].

Garmi et al. showed, in vitro, that an induced sharp decidual incision, imitating the in vivo process, that is, cesarean section, increased significantly the invasion potential of the trophoblastic cells. Additionally complete reapproximation of the incised edges of the decidua in vitro made the incised decidua to behave similarly to intact decidua while restricting once again the extent of the invasiveness. Using the same cohort of trophoblast cells, while changing only the decidual anatomic characteristics', the invasion potential of trophoblastic cells in vitro changed accordingly emphasizing the role of decidua on the invasion potential [15].

## 4. Diagnosis

Placenta accreta should be suspected in women who have both a placenta previa, particularly anterior, and a history of cesarean or other uterine surgery. The most important factor affecting outcome is prenatal diagnosis of this condition. It gives the opportunity to make a delivery plan that properly anticipates the expected blood loss and other potential complications of delivery. In addition, it gives the opportunity for electively timing the procedure since prevention of complications ideally requires the presence of a multidisciplinary surgical team.

Antenatal ultrasound is the technique of choice used to establish the diagnosis and guide clinical management [17]. Signs of accretion may be seen as early as in the first trimester. Comstock retrospectively reviewed the ultrasound examinations performed up to 10 gestational weeks among women later proven to have placenta accreta on pathological examination. All had low-lying gestational sacs which are clearly attached to the uterine scar. The myometrium was thin in the area of the scar to which the sac was attached compared to normal early gestational sacs [30].

Though the predictive value of first trimester ultrasound for this diagnosis remains unknown, since most reports are based on isolated case reports, still, women with signs of accretion in the first trimester should undergo follow-up imaging later in the second and third trimester with attention to the potential presence of placenta accreta [31].

Second and third trimester gray-scale sonographic characteristics include loss of continuity of the uterine wall, multiple vascular lacunae (irregular vascular spaces) within placenta, giving “Swiss cheese” appearance adjacent to the placental implantation site, lack of a hypoechoic border (myometrial zone) between the placenta and the myometrium, bulging of the placental/myometrial site into the bladder, and increased vasculature evident on color Doppler sonography. Wong et al. reported that using a composite scoring system of 6 sonographic findings performed with gray-scale and Doppler sonography had 89% sensitivity and 98% specificity for the diagnosis of placenta accreta [32]. Shih et al. compared three-dimensional power Doppler with gray-scale and color Doppler ultrasonography for diagnosing placenta accreta. Three-dimensional power Doppler was targeted to detect angioarchitecture in the basal and lateral views of the placenta. The ultrasound findings were analyzed with reference to the final diagnosis made during Cesarean delivery. The authors observed that “numerous coherent vessels” detected by three-dimensional power Doppler in the basal view were the best single criterion for the diagnosis of placenta accreta, with a sensitivity of 97% and a specificity of 92%. They concluded that three-dimensional power Doppler may be useful as a complementary technique for the antenatal diagnosis or exclusion of placenta accreta [33].

If the ultrasound findings are not considered definitive, or the placenta is located on the posterior wall, magnetic resonance imaging can be performed using gadolinium contrast intravenously. Magnetic resonance imaging findings considered suspicious for the presence of placenta accreta include placental heterogeneity, mass effect of the placenta into the underlying bladder or extending laterally or posteriorly beyond the normal uterine contour, obliteration of the myometrial zone visible on initial uptake of gadolinium, and a beading nodularity within the placenta [34].

Other than the imaging methods, elevated biochemical markers in maternal serum such as elevated levels of alpha fetoprotein and human chorionic gonadotropin within the triple screening test have been reported to be associated with an increased risk of placenta accreta. Though the mechanism is unclear, abnormality of the placental-uterine interface that may lead to leakage into the maternal circulation may explain this increase [35, 36].

At this time no antenatal diagnostic technique affords the clinician 100% assurance of either ruling in or ruling out the presence of placenta accreta. The definitive diagnosis of placenta accreta is usually made postpartum on hysterectomy specimens when an area of accretion shows chorionic villi in direct contact with the myometrium and absence of decidua [26, 37].

## 5. Management

Women with placenta accreta are usually delivered by a cesarean section. It is better to perform the surgery under elective, controlled conditions rather than as an emergency without adequate preparation. In addition, regardless of the management option made, prevention of complications ideally requires a multidisciplinary team approach [9]. The multidisciplinary team should include a gynecologic surgeon experienced in pelvic surgery, a blood bank team prepared to administer multiple blood components, experienced anesthesiology personnel who are skilled in obstetric anesthesia, skilled urologists in case a bladder resection or repair might be required, experienced intensivists for postpartum care, and an experienced neonatologist. In cases where pelvic artery catheterizations are used, an experienced interventional radiologist is also required. Additionally, Eller et al. showed that delivery at a medical center with a multidisciplinary care team resulted in a more than 50% risk reduction for composite early morbidity among all cases of placenta accreta and a nearly 80% risk reduction among those cases wherein accreta was suspected before delivery [38].

There is a great benefit of planned as opposed to emergent peripartum hysterectomy. In mothers with placenta previa and a suspected accreta who required peripartum hysterectomy, a scheduled delivery has been associated with shorter operative times and lower frequency of transfusions, complications, and intensive care unit admissions [39].

Accordingly timing of delivery may have a crucial impact on maternal and perinatal outcome. O'Brien et al. reported that after 35 weeks, 93% of patients with placenta accreta experience hemorrhage necessitating delivery [5]. Additionally Warshak et al. reported that planned delivery at 34 to 35 weeks of gestation in a cohort of 99 cases of accreta did not significantly increase neonatal morbidity [9]. Robinson and Grobman compared strategies for the timing of delivery in individuals with placenta previa and ultrasonographic evidence of placenta accreta to determine the optimal gestational age for delivery. The strategies ranged from a scheduled delivery at 34, 35, 36, 37, 38, or 39 weeks of gestation to a scheduled delivery at 36, 37, or 38 weeks of gestation only after amniocentesis confirmation of fetal lung maturity. They found that a scheduled delivery at 34 weeks of gestation was the preferred strategy and that at any given gestational age, incorporating amniocentesis for verification of fetal lung maturity does not assist in the management of such individuals [39]. In view of that and in order to avoid an emergency cesarean on the one hand and to minimize complications of prematurity on the other, it is acceptable to schedule cesarean at 34 to 35 weeks.

The best anesthetic method among women with placenta accreta is controversial. The American Society of Anesthesiologists task force on obstetric anesthesia suggested that general anesthesia may be the most appropriate choice in some circumstances, including cases where severe hemorrhage is anticipated [40]. Chestnut et al. suggested that epidural anesthesia might be an appropriate choice for some of these patients. However, the decision to administer regional anesthesia should be individualized and made only after review of the pertinent history, physical examination, and appropriate laboratory/imaging data. Extensive pelvic invasion and/or significant potential for major intraoperative bleeding still favors general anesthesia [41].

Regardless of the anesthetic technique used, anesthesia considerations should include insertion of large-bore venous access to allow rapid crystalloid and blood product infusion, availability of high flow rate infusion and suction devices, hemodynamic monitoring capabilities (central venous and peripheral arterial access), compression stockings and devices to prevent thromboembolism, padding and positioning to prevent nerve compression, and avoidance and treatment of hypothermia [31]. Consideration may be given to the use of the cell saver and acute normovolemic hemodilution. While both these techniques remain controversial for the parturient, recent data attest to their safety and efficacy [42].

Placenta accreta is most likely to affect the urinary bladder [23]. Placenta accreta that invades the urinary bladder may cause urinary fistula, ureteral transection, and bladder laceration requiring partial or total cystectomy [23]. Data suggest that preoperative ureteric stent placement may help reduce the risk of ureteric injury. Moreover, cystoscopic placement of ureteric stents can usually be accomplished quickly and easily even in an emergency and is associated with relatively minimal risk [43]. If bladder involvement is suspected, cystotomy may be needed to clarify the extent of invasion after devascularization of the uterus is achieved and to ensure ureteric patency if stents were initially not inserted [31].

The optimal management after delivery of the neonate is vague. The literature is based on small case reports and retrospective analysis but lacks prospective trials. Hysterectomy immediately after delivery of the neonate without attempts at placental removal had been reported to lower mortality and morbidity rates compared to conservative management especially in cases of placenta percreta. This procedure became, and still is, since 1972, the recommended treatment option [18, 19, 44].

Conservative management, which includes delivery by a cesarean section without hysterectomy, had been proposed in selective cases to preserve fertility. The primary idea of conservative management is to leave the entire placenta or just the part that is adherent to the myometrium in situ and to preserve the uterus. Manual removal of densely adherent placental areas should not be tried because forceful separation may result in severe bleeding [20, 45]. Kayem et al. compared maternal outcomes among women with a placenta accreta, within two consecutive periods: period A, the placenta was removed manually leaving the uterine cavity empty; period B, the placenta was left in situ. During period B, there was a significant reduction in the hysterectomy rate, the mean number of red blood cells transfused, and in the incidence of disseminated intravascular coagulation compared with period A [46].

Postoperative complications reported with a conservative approach include severe postpartum hemorrhage, postoperative disseminated intravascular coagulopathy, and infection resistant to antimicrobial therapy that may require laparotomy and hysterectomy [17, 21, 47, 48]. Timmermans et al. reviewed all articles on conservative management of abnormally invasive placentation published from 1985 through 2006. During the study period, 48 reports described outcomes of 60 women who were treated conservatively for abnormally invasive placentation. Preserving the uterus succeeded in about 80% of the women with subsequent pregnancies in at least approximately one-sixth of women. Treatment failure due to vaginal bleeding was the most important complication of conservative management of abnormally invasive placentation requiring prolonged follow-up. The authors concluded that conservative management of abnormally invasive placentation can be effective and fertility can be preserved. However, it should only be considered in highly selected cases when blood loss is minimal and there is a desire for fertility preservation [21].

Another retrospective, multicenter study of conservative management of placenta accreta reported the outcome of 167 women treated in 25 French university hospitals. The authors showed that in centers with adequate equipment and resources, conservative treatment for placenta accreta is a valuable option with a success rate of 78.4% and a severe maternal morbidity rate of 6.0%. However, conservative treatment required women adherence to treatment over a long postpartum period, which suggests that women may continue to be at risk for severe morbidity and possibly mortality for weeks or even months after delivery. In view of that and until randomized trials are performed, the authors suggested that cesarean hysterectomy without attempt of placental removal should be strongly considered for placenta accreta in multiparous women not interested in preserving their fertility according to The authors [49]. In a subsequent study, the same group described the fertility and pregnancy outcomes after successful conservative treatment for placenta accreta, that is, uterine preservation. Of all 96 women available for follow-up 8.3% had severe intrauterine synechiae and were amenorrheic. Of the 27 women who desired more children, 88.9% had had 34 pregnancies that resulted in 21 deliveries of healthy babies born after 34 weeks of gestation. The mean time to conception was 17.3 months. Placenta accreta recurred in 28.6% of cases [50].

In spite of the described outcomes, it is worth to mention the limitations of review of case reports that occasionally may represent a publication bias. Lethal and other severe complications of conservative management of placenta accreta may be scarcely reported and prone to being underreported while good outcomes may be overreported. Additionally, the wide variety of management techniques makes it hazardous to draw strong conclusions on effectiveness.

The role of adjuvant methotrexate in cases of conservative management is uncertain. No large studies have compared methotrexate with no methotrexate in the treatment of placenta accreta, and at the present time, there are no convincing data for or against the use of Methotrexate in cases of placenta accreta [21].

Recently, inserting intravascular balloon catheter for occlusion and/or arterial embolization of the pelvic arteries was introduced as an invasive adjuvant therapy in order to minimize blood loss during cesarean hysterectomy. In selective cases the placement of a balloon catheter was done concurrently with conservative management with the intent of avoiding hysterectomy, thereby preserving fertility [51]. Placement of intravascular balloon catheters has been performed at various sites from as proximal as the aorta [52] to more distally within the anterior division of the internal iliac arteries [53]. More often than not, this technique has been combined with concomitant arterial embolization. The rational of inserting intravascular balloon catheters is to decrease blood flow to the uterus and potentially lead to reduced blood loss. In addition it makes possible to perform surgery under easier, more controlled circumstances, with less profuse hemorrhage. Thus far, the results of using preoperative prophylactic internal iliac artery catheterization as an adjuvant treatment to hysterectomy or in cases of conservative management are equivocal and are largely limited by the small sample size. Though several studies have shown that preoperative prophylactic artery catheterization may reduce intraoperative blood loss and transfusion requirements in patients with placenta accreta or may assist in preserving fertility [1, 14, 53–55], others did not show that its use was beneficial for women with placenta accreta. Additionally the procedure is not without harm and may be accompanied with other vascular complications [21, 56–58].

Shrivastava et al. suggested that failure of intravascular balloon catheters to reduce blood loss may be explained by the extensive degree of uterine blood flow with pregnancy and the extensive vascular anastomoses present in the gravid pelvis. In addition, whereas reduction of blood flow to the uterine arteries likely occurs following balloon inflation in the hypogastric arteries, collateral circulation from cervical, ovarian, rectal, femoral, lumbar, and sacral arteries likely contribute to the overall blood loss. Inflation of the balloons immediately following delivery of the infant may actually exacerbate collateral blood flow [57].

## 6. Conclusion

Placenta accreta is becoming an increasingly common complication of pregnancy. Prenatal diagnosis seems to be a key factor in optimizing the counseling, treatment, and outcome of women with placenta accreta. Cesarean hysterectomy is probably the preferable treatment.

Conservative management should only be used in highly selected cases. Even though there may be a rationale to add adjuvant therapy in such cases, there is no evidence-based proof that such therapy is actually of benefit or that it is not in fact harmful.
